# Exposure of biosynthesized nanoscale ZnO to *Brassica juncea* crop plant: morphological, biochemical and molecular aspects

**DOI:** 10.1038/s41598-020-65271-y

**Published:** 2020-05-22

**Authors:** Jahirul Ahmed Mazumder, Ehasanullah Khan, Mohammad Perwez, Meetu Gupta, Sanjay Kumar, Khalid Raza, Meryam Sardar

**Affiliations:** 1 Department of Biosciences, Jamia Millia Islamia, New Delhi, 110025 India; 2 Department of Biotechnology, Jamia Millia Islamia, New Delhi, India; 3 Department of Computer Science, Jamia Millia Islamia, New Delhi, India

**Keywords:** Biochemistry, Biotechnology, Plant sciences, Nanoscience and technology

## Abstract

The present work describes the *in vitro* synthesis and characterization of Zinc oxide nanoparticles (ZnO NPs) using an enzyme alpha amylase, the synthesized nanoparticles were used to study their beneficial effect in the growth and development of *Brassica juncea*. Transmission Electron Microscope (TEM) image reveals the average size of ZnO NPs was 11 nm and X-ray powder diffraction (XRD) suggests nanoparticles were crystalline in nature. In-silico study confirmed lysine, glutamine and tyrosine present in alpha amylase enzyme, plays a crucial role in the reduction of Zinc acetate dihydrate to ZnO NPs. The biochemical parameters and oxidative enzymes of *Brassica juncea* were compared with ZnO NPs treated plants. The effect of ZnO NPs on the cellular expression of metal tolerant protein (*BjMTP*) and cation efflux transporter gene (*BjCET2*) was also studied. The results indicate that nanoparticles can be used as a replacement for traditional harmful chemical fertilizers.

## Introduction

Particles of size less than 100 nm are characterized as nanoparticle, these are formed when elements are modified by altering at their molecular and atomic level^1^. This branch of science has brought a boon in scientific research by introducing various nano sized particles that have importance in various fields such as medicine, technology, agriculture etc. Nanoparticles show size dependent electronic, optical and chemical properties. Among the different nanomaterials metal based nanoparticles are the most studied nanomaterials^2^. A characteristic surface plasmon resonance (SPR) absorption in the UV Visible region is a unique property of metal nanoparticles. The SPR band arises due to the presence of free electrons in the conduction band^3^.

Nanotechnology has shown numerous beneficial effects in the field of agriculture^4^. Several investigators reported the beneficial and harmful effect of nanomaterial’s on plant growth and a number of review articles have been published on the interaction mechanism of plants and nanomaterials^5,6^. These interactions may results in physiological, morphological, and genotoxic changes in plants, basically their mechanism is important for their effective use in agriculture. Nanoparticles increase the productivity of plants by interacting directly with the plants or indirectly by interacting with the soil. They increase the growth of plants by site-targeted controlled delivery of nutrients, or by maintaining the level of micronutrients, they also provide resistance to the plants by their action on phytopathogens^7^. Nanomaterial’s improve soil health by chelating various ions/ salts present in soil, regulate the pH of soil, and also interact with the microbes present in the soil, thus indirectly help the plant growth^8–10^. Metal Nanoparticles like Ag, Au, TiO_2_, ZnO, iron etc have been reported to stimulate plant growth^11^. Among these Zinc Oxide nanoparticles (ZnO NPs) have been widely used, as zinc is an essential micronutrient and participates in various metabolic reactions^12^. Several scientists studied the effect of ZnO NPs on different crop plants and their studies reveal that ZnO NPs exerts positive effect on the plant growth. It is also reported that the toxicity of ZnO NPs on crop plants is much lower as compared to the toxicity of Zn^2+^ or ZnO bulk particles^13^.

Nano fertilizers like Nano-GroTM, Nano-Ag Answer^R^, TAG NANO (NPK, PhoS, Zinc, Cal, etc.) are marketed by some commercial companies^14^, however, there is a need for large-scale industrial production of nanofertilizers and nanopesticides^15^. In developing countries like India, 18% of India’s gross domestic product (GDP) depends on agricultural sector and provides 50% employment, thus research is required to synthesize nanoparticles which can improve the crop qualities and their yield^16^. There are many methods identified for the preparation of metal nanoparticles, namely physical, chemical and biological methods. Physical preparation of nanoparticles is expensive and consumes enormous energy whereas chemical method involves toxic chemicals. Biological synthesis involves the use of biomolecules, plant extract, natural compounds and microbes etc as reducing agents for reduction of metal salt into nano formulation. Among various biomolecules, enzymes have the advantage that they can act as a reducing as well as capping agent, thus synthesis can be achieved in a single step. Another very important feature of the enzymes is that they can operate outside the cell also, which makes them suitable for biotechnological applications. Moreover, the biological approach is considered safe and as better alternative to chemical and physical methods. Many research papers have been published on biological synthesis of nanomaterial’s but synthesis of nanoparticles using enzymes is still an unexplored area^17^.

Therefore, in the present paper ZnO NPs were synthesized *in vitro* using an enzyme alpha amylase and the effect of biosynthesized nanoparticles were studied on an industrially/agriculturally important crop *Brassica juncea*. This is widely cultivated as a leaf vegetable, a root vegetable, and an oilseed. Indian mustard, *Brassica juncea (L)* is one of the most important edible oil seed crops of the Indo-Gangetic plains. This crop is also used in phytoremediation to remove heavy metals, such as lead and cadmium from soil^18,19^. Because of acute shortage of oilseed in India, it is being imported from different parts of the world. The shortage of oilseed such as *Brassica* is due to a number of reasons primarily being deficiency of micronutrient, lands with soils of poor fertility, pests and diseases also cause substantial production losses^20–22^.

## Results

**Figure 1:**
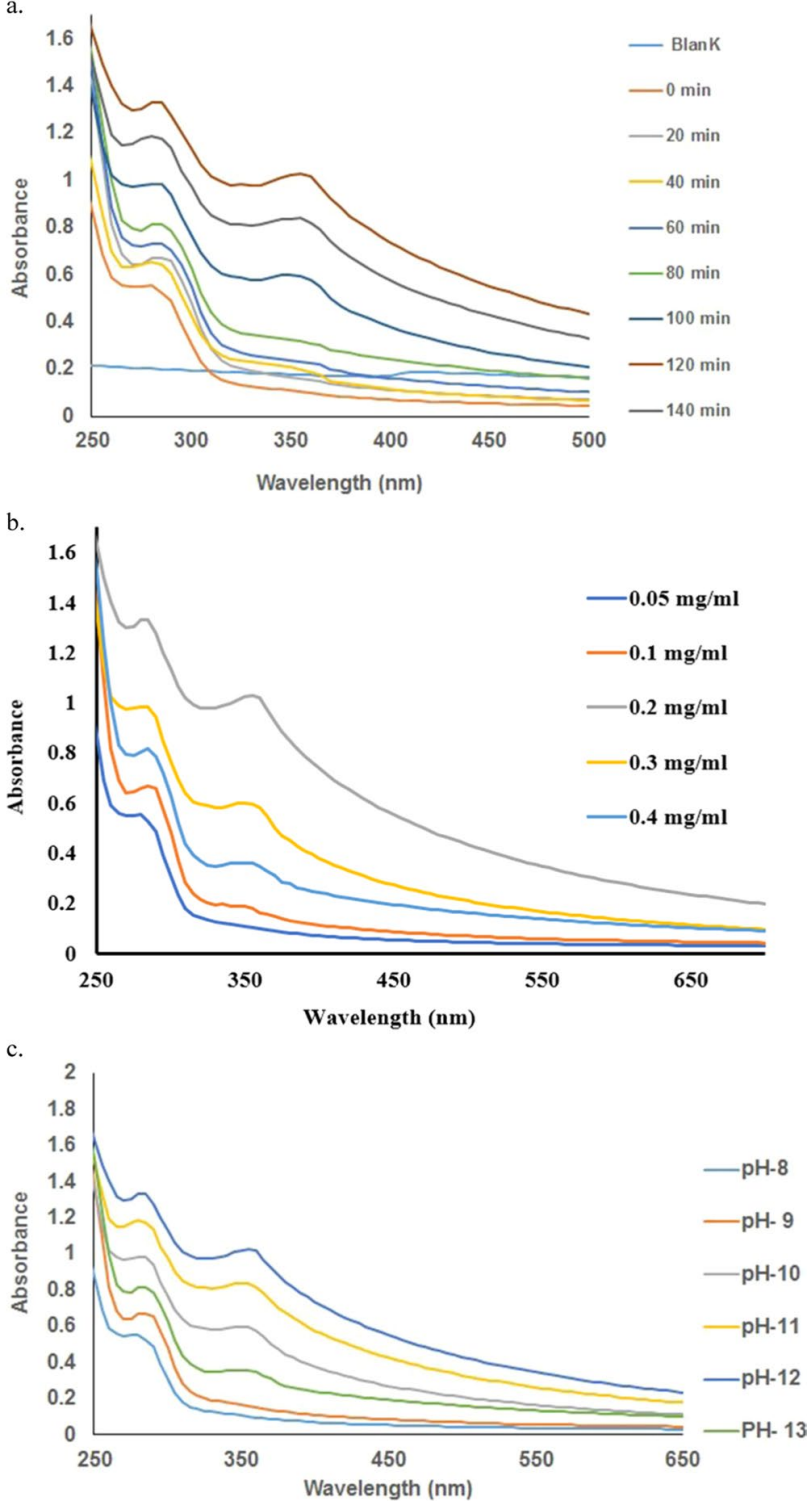
UV-Vis spectra of ZnO nanoparticles: (**a**) Recorded at a function of time, 0.02M Zinc acetate dihydrate was considered as blank. (**b**) At different concentration of alpha amylase enzyme. (**c**) At a function of pH. Enzyme concentration for the synthesis was kept at 0.2 mg/ml.

Enzyme mediated synthesis is considered eco-friendly and biocompatible; it is a one-step reaction where the enzyme plays the role of catalyst as well as stabilizer^23^. Although enzyme mediated synthesis has several advantages but not much research has been done on this aspect. Recently, we reported the *in vitro* synthesis of silver and gold nanoparticles using the pure enzyme Alpha amylase^24,25^, a digestive enzyme that catalyses the hydrolysis of starch into simple sugars and this enzyme is easily available at low price. To further explore the possibility of using this enzyme for synthesis of other metal nanoparticles, here we studied the role of this enzyme in the synthesis of ZnO NPs. When alpha amylase enzyme solution was incubated with aqueous solution of Zinc acetate dihydrate at 25 °C, the colourless solution turns off white in 2 hours indicating formation of ZnO NPs, which was observed visually. The UV-Vis spectra of ZnO NPs (Fig. 1a) depicts that the SPR band of ZnO NPs occurs at 364 nm, the second absorption band observed at 280 nm was due to the enzyme alpha amylase. The blank (Zinc acetate dihydrate solution) did not show any change in colour with time, which suggests that alpha amylase is responsible for the synthesis of ZnO NPs. The performance parameters like pH, concentration of enzymes, Zinc acetate dihydrate salt were also investigated to get the optimum synthesis. Figure 1b shows that the optimum concentration of alpha amylase for the synthesis is 0.2 mg/ml, (20U), to study the effect of pH, synthesis was carried out at different pH (8–12), and the optimum pH was observed at 12 (Fig. 1c). Several investigators have reported that synthesis of ZnO NPs is pH dependent, also the size of ZnO NPs varies with the change in pH value^26,27^. At pH 6-7, the particle agglomerates due to acidic and neutral pH values of Zn(OH)_2_ sols during synthesis. OH^-^ ions are required for the ZnO conversion, as it allows nucleation, growth and particle formation, pH 6 and 7 lacks OH^-^ ions. With the increase in pH, agglomeration rate decreases and particle size becomes smaller due to the alkaline condition^28^. Other researchers have also reported that the concentration of reducing agent, metal salt, pH, temperature plays an important role in synthesis^29–31^.

**Figure 2:**
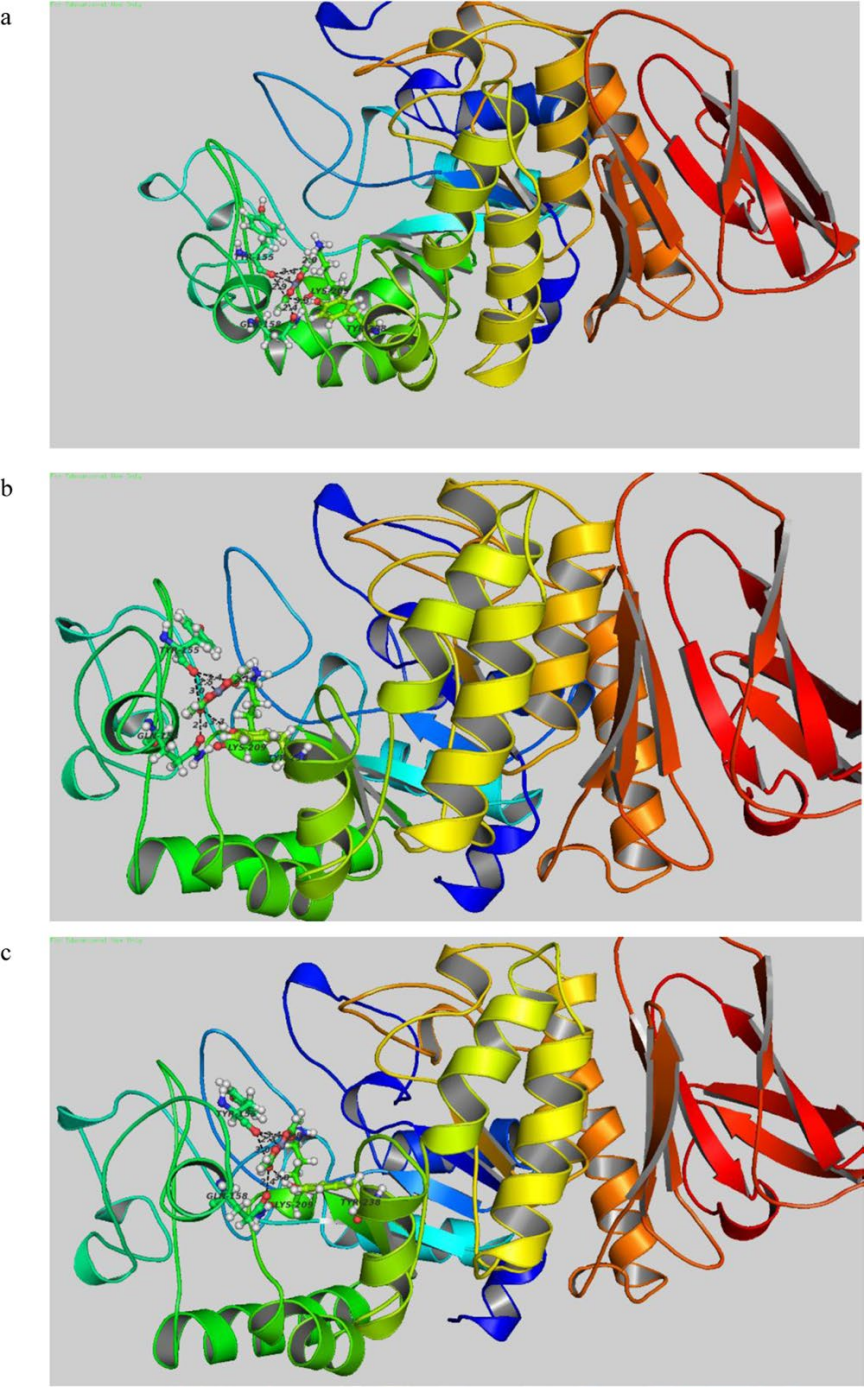
Structural view of docking between alpha-amylase and Zinc acetate (**a**) first/best scoring result with respective residues involved and distance of separation. (**b**) Second top scoring result. (**c**) Third top scoring result.

The stability of these nanoparticles was also studied by keeping them at room temperature for six months and taking the UV spectra (data not shown), no change in the SPR was observed indicating the nanoparticles were quite stable. To study the mechanism of synthesis of ZnO NPs with alpha amylase, in-silico studies using GOLD 5.2.2 was carried out. The studies revealed the possible protein-ligand interactions and the amino acids with their respective distance responsible for the conversion of Zinc acetate dihydrate[(CH_3_COO)_2_Zn.2 H_2_O] to ZnO NPs (Fig. 2a–c). Number of interactions determined through docking between alpha-amylase and Zinc acetate with their respective type of interaction has been summarized in the Table 1 for loop segment. Studies indicate lysine(209), glutamine(158) and tyrosine(155, 238) present in the binding site interact with the Zinc acetate and plays crucial role in the conversion of Zinc acetate to ZnO NPs. The protein ligand interaction showed that the responsible amino acids were all same at about same place/ cavity with different distance of interactions each time. Interaction of ZnO NPs using lysine and glutamine amino acid has been reported earlier also^32,33^.

**Table 1:** Number of interactions in the binding site determined through docking between α-amylase and Zinc acetate with their respective type of interaction.

Index	Entry	Gold score. Fitness	Interacting residues	Ionic/Hydrogen bond interactions number
1	Structure2D_CID_11192 | dock1|	36.2783	Tyr155, Gln158, Lys209, Tyr238	6
2	Structure2D_CID_11192 | dock3|	36.0536	Tyr155, Gln158, Lys209, Tyr238	6
3	Structure2D_CID_11192 | dock2|	35.4436	Tyr155, Gln158, Lys209, Tyr238	6

**Figure 3:**
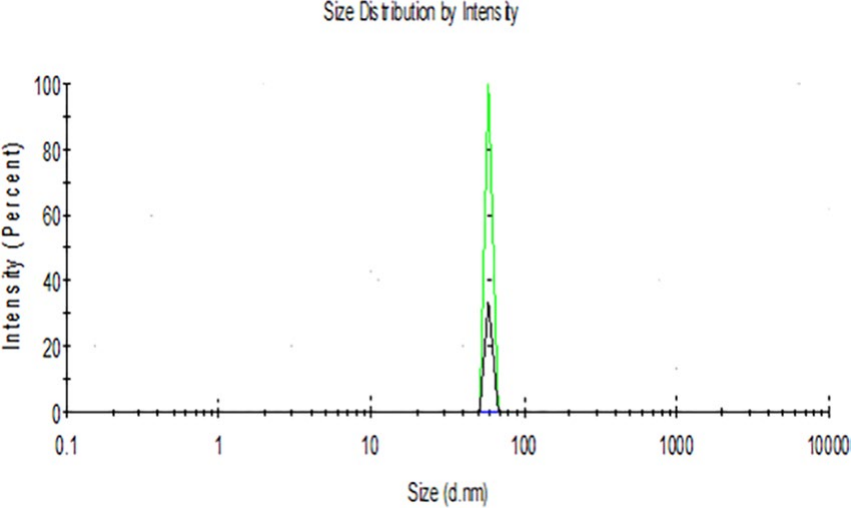
Dynamic Light scattering image showing mean average size of ZnO NPs.

**Figure 4:**
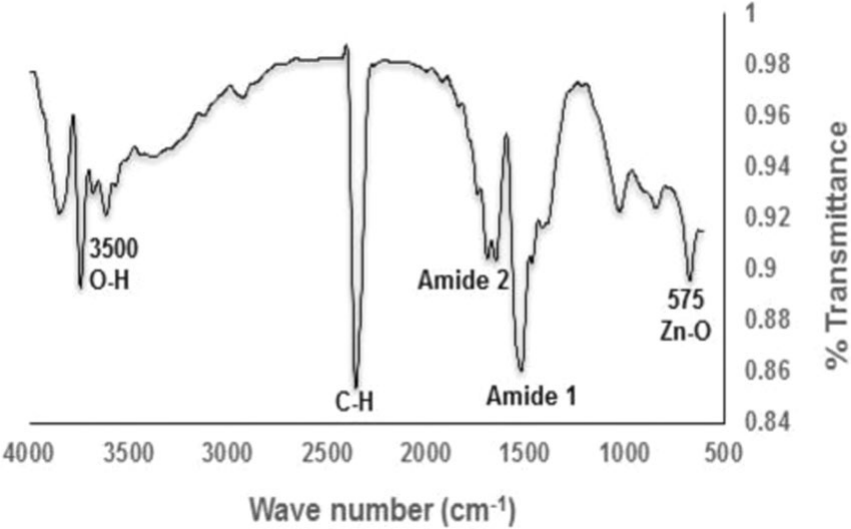
Fourier-transform infrared (FTIR) spectra of ZnO NPs.

**Figure 5:**
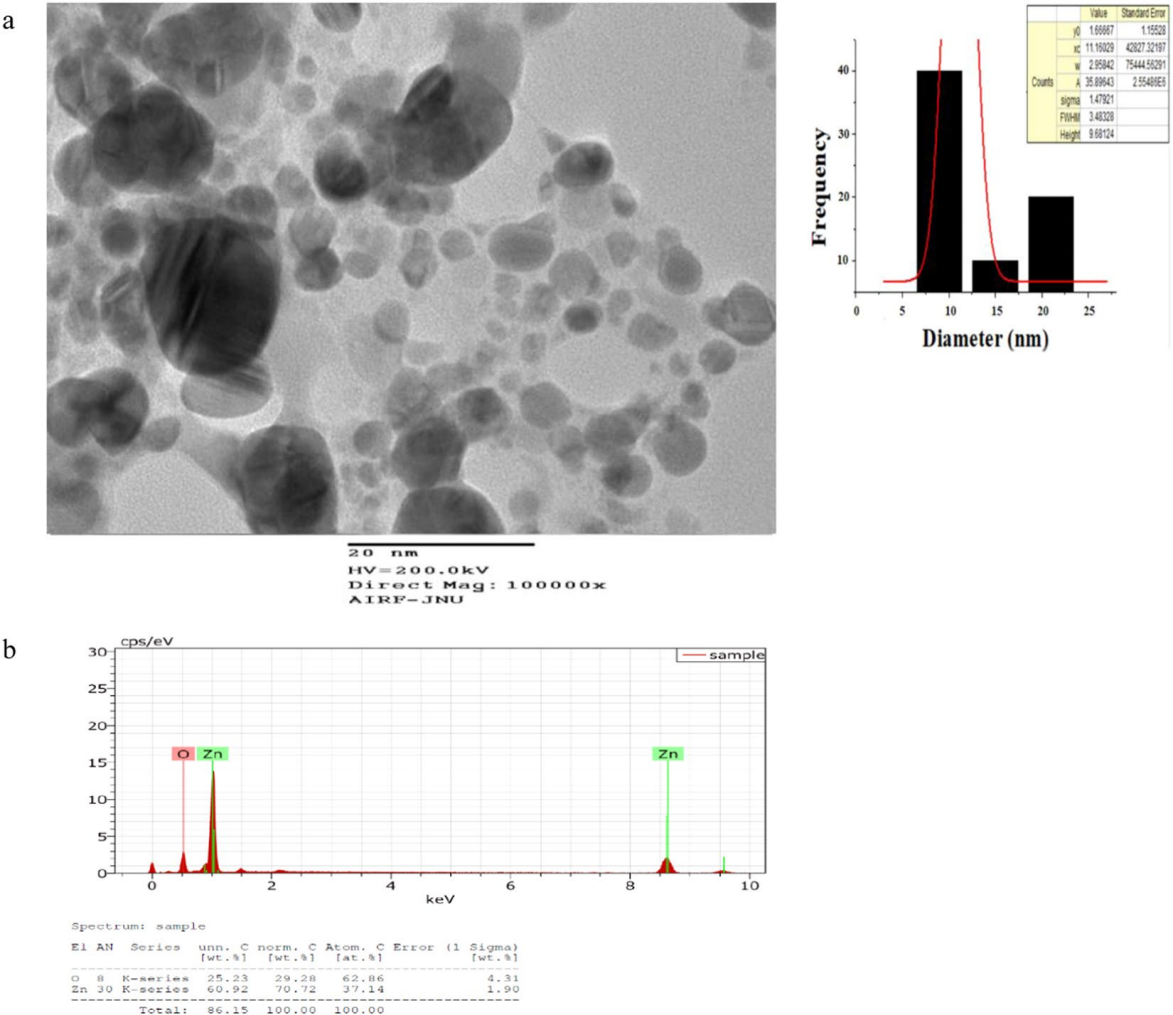
(**a**) Transmission electron microscopy image of ZnO NPs. In size distribution curve ‘xc’ denotes mean particle size which is 11 nm and are spherical in shape. (**b**) Energy-dispersive X-ray spectroscopy of ZnO nanoparticles.

**Figure 6:**
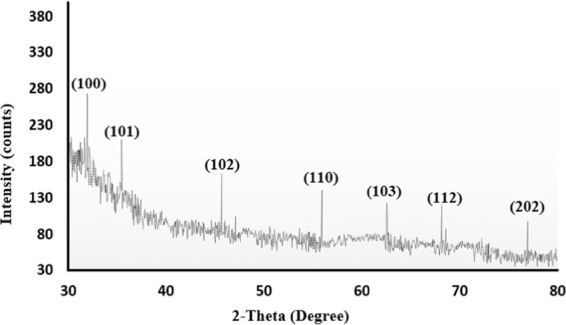
X-ray diffraction pattern of ZnO NPs synthesized using alpha amylase.

**Figure 7:**
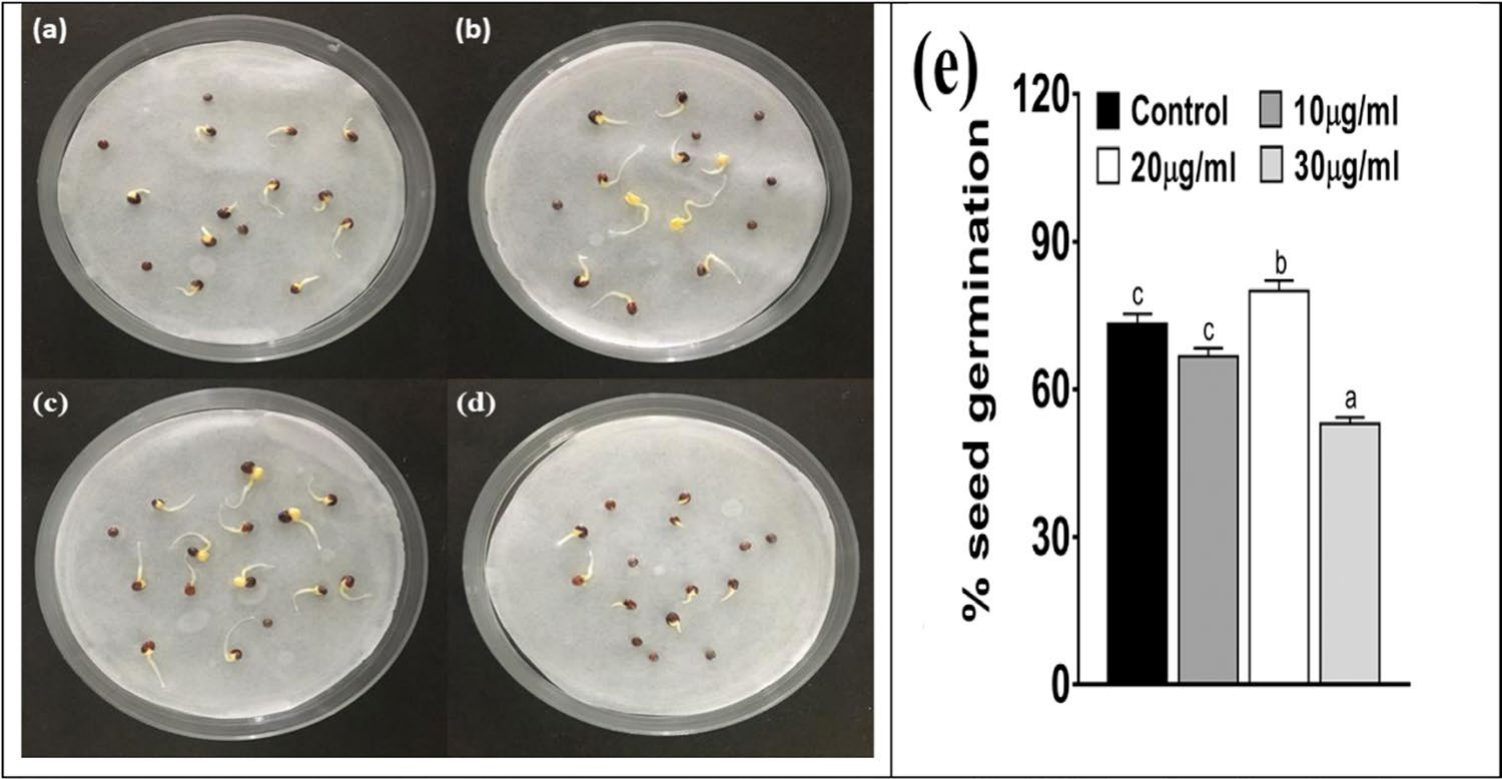
Change in percent seed germination under different concentration of ZnO NP. (**a**) Control (only 5% HM), (**b**) Seeds treated with 10 μg/ml ZnO NPs, (**c**) Seeds treated with 20 μg/ml ZnO NPs, (**d**) Seeds treated with 30 μg/ml ZnO NPs. (**e**) Different letters (a, b and c) indicates significant different between each treatment at p < 0.05. Values are means of 5 replicates (n = 5).

**Figure 8:**
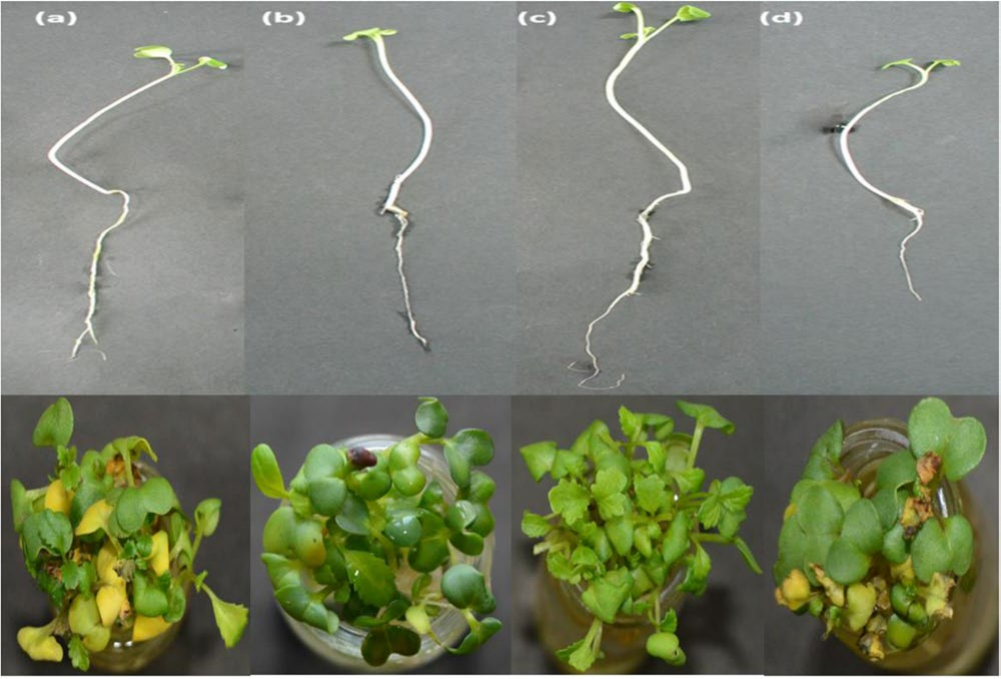
Phenotypic changes in plants grown in different conditions along with 10% HM. (**a**) Control, (**b**) Test 1 (10 μg/ml ZnO NPs solution), (**c**) Test 2 (20 μg/ml ZnO NPs solution), (**d**) Test 3 (30 μg/ml ZnO NPs solution).

**Figure 9:**
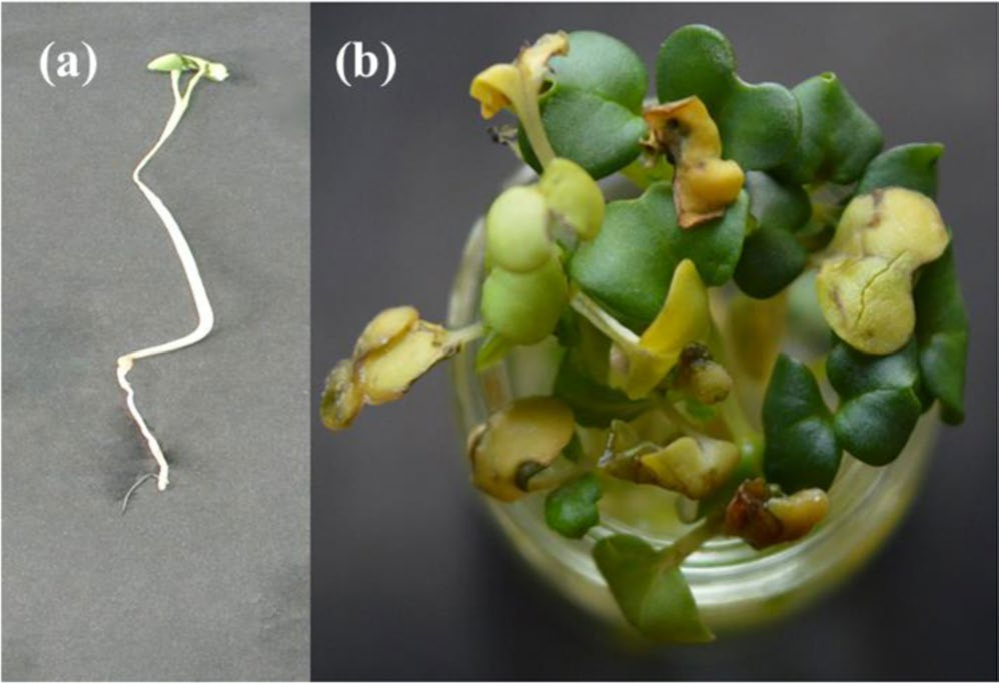
(**a**) Image of plant grown in Zinc acetate dihydrate (20 μg/ml). (**b**) Plants with dried out and yellow leaves.

Reduction of [(CH_3_COO)_2_Zn.2H_2_O] to ZnO NPs in presence of reducing agent [lysine(209), glutamine (158) and tyrosine(155, 238) present in the binding site of alpha amylase] can be represented as follows:

DLS image of ZnO NPs depicts that the size of nanoparticles in colloidal solution is approximately 70 nm (Fig. 3). To know the particle size distribution of the ZnO NPs, Polydispersity index (PDI) was determined and was found to be 0.39. The PDI remains same even after 6 months. Zeta potential was used to find the stability of colloidal solution and was found to be −28.2 mV ± 4.48 mV(Standard Deviation), which indicate stability of the colloidal solutions. FTIR spectrum (Fig. 4) reveals sharp peak at 3500 cm^−1^, 1650 cm^−1^, 1540 cm^−1^ and at 575 cm^−1^. The peak at 3500 cm^−1^ is due to O–H stretching of water. The peak at 1650 cm^−1^ and 1540 cm^−1^ is due to N-H stretching of amide bond of proteins. The peak at 575 cm^−1^ is due to the stretching of Zn with oxygen^34^. The morphology of the ZnO NPs were studied with the help of TEM. Figure 5 a revealed the particles were spherical in shape and size distribution curve ‘xc’ denotes mean particle size which is 11 nm, EDX profile shows strong zinc and oxygen signal (Fig. 5b). XRD pattern of alpha amylase assisted synthesis of ZnO NPs is shown in (Fig. 6). XRD of purified powdered ZnO NPs have peaks at [1 0 0], [1 0 1], [1 1 0], [1 1 2] and [2 0 2] which closely relates with the standard value of JCPDS data card no. 36–1451^35^. Further the synthesized ZnO NPs were used to study their effect on the growth of *Brassica juncea L*. The sterilised seeds (15 nos) of *Brassica* plants after soaking overnight were treated with different concentration of ZnO NPs (10–30 μg/ml) and after 3 days, the germination rate was monitored. In control (without ZnO NPs), 73% ± 2 (11 out of 15), in test (treated with 10 μg/ml ZnO NPs), 66% ± 1.7 (10 out of 15), test plant that was treated with 20 μg/ml ZnO NPs showed 80% ± 2.1 (12 out of 15) germination, and finally the test plant that was treated with and 30 μg/ml ZnO NPs showed poor rate of germination, only 53% ± 1.3 (8 out of 15) as shown in Fig. 7a–e. The germinated seeds were then transferred to the growth chamber, the length of root and shoot in the test plant and Control were measured after 7^th^ day. The results indicate that root and shoot length were significantly more in the plants treated with 20 μg/ml of ZnO NPs as compared to the control plant, shown in Fig. 8a–d. The least length was observed in plants treated with 30 μg/ml of ZnO NPs, shown in Fig. 8d. To study the effect of Zinc acetate salt and alpha amylase enzyme on plant growth, two separate experiments were also run, for experiment 1, fresh plants were treated with Zinc acetate salt solution (20 μg/ml in HM) and for experiment 2 fresh plants were treated with alpha amylase enzyme (1 mg/ml in HM). In experiment 1, the leaves of the plants that were treated with Zinc acetate solution became yellow and started drying out as shown in Fig. 9a,b. No change in growth rate was observed among the plants treated with alpha amylase enzyme solution in experiment 2, these two experiments suggested that only ZnO NPs is responsible for the better growth of *Brassica* plant. The result indicating that 20 µg/ml ZnO NPs enhanced seed germination and plant height, however, 30 µg/ml or higher concentration showed significant decrease in seed germination and plant height which indicating that ZnO NPs up to 20 µg/ml is beneficial for plant and can be used as fertilizers.

**Figure 10:**
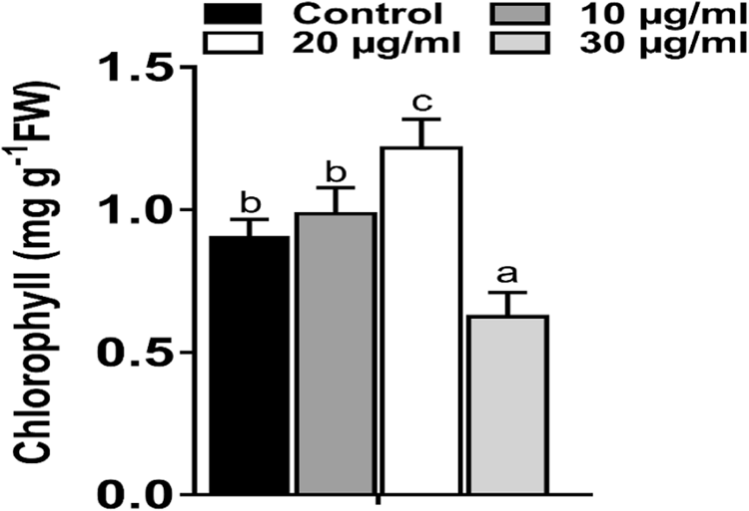
Effects of different concentration of ZnO NP on total chlorophyll. (**a**) Control (only 5% HM), (**b**) Seeds treated with 10 μg/ml ZnO NPs, (**c**) Seeds treated with 20 μg/ml ZnO NPs, (**d**) Seeds treated with 30 μg/ml ZnO NPs. (**e**) Different letters (a, b and c) indicates significant different between each treatment at p < 0.05. Values are means of 5 replicates (n = 5).

**Figure 11:**
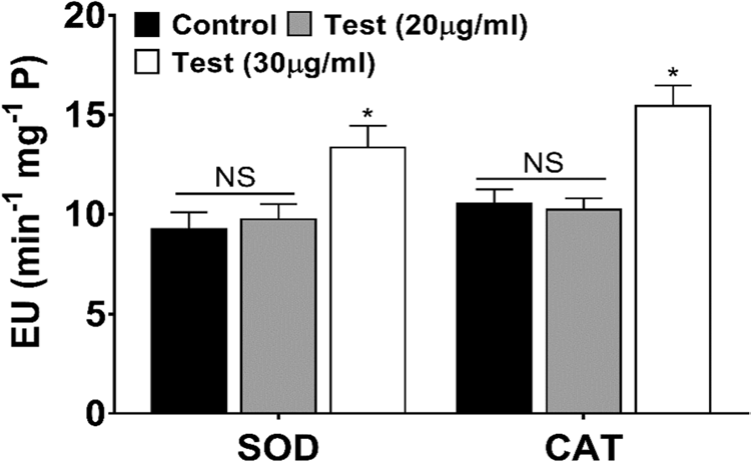
Activity of Superoxide dismutase (SOD) and Catalase (CAT) at 20 μg/ml and 30 μg/ml ZnO NP. NS represents non-significant difference, whereas * represents significant difference as compared to control. Values are means of 5 replicates (n = 5).

Further to confirm the effect of ZnO NPs on the leaves of *Brassica*, chlorophyll content (Chlorophyll a + b) was determined. The plants treated with ZnO NPs (20 μg/ml) have high chlorophyll content (34%) as compared to control, as shown in Fig. 10. The most common effects of excessive zinc uptake are, loss of chlorophyll content in plants^36^, chlorosis of the newer leaves^37^, necrotic leaf tips^37^, retarded growth of the entire plant, and/or reduced root growth^38^. The result indicating that plant supplied with 20 µg/ml ZnO NPs may be beneficial for sustainable growth and development. On the other hand, 30 µg/ml ZnO NPs showing marked decrease in chlorophyll content (30% less as compared to control), reduced germination rate as well as root and shoot growth, thus suggesting the negative involvement of ZnO NPs (when present in excess) in chlorophyll biosynthesis. Zn is an essential micro nutrient to plant, however, in excess it can be lethal for plant; there are several reports which has shown that Zn at higher concentration increase the production of reactive oxygen species that causes oxidative stress which can leads to the cell death^39^. Plants in response to the excessive ROS (reactive oxygen species) production enhance the production/activity of non-enzymatic and enzymatic (i.e. SOD and CAT) antioxidant which control the ROS level. SOD and CAT activities were analysed in leaf of *Brassica* plant as shown in Fig. 11. The result indicated the nonsignificant change in activity of both SOD and CAT in 20 µg/ml treated plants compared to control. However, in 30 µg/ml treated plants, the activity of both SOD and CAT increased significantly (p < 0.05) by 44.43% and 46.72% respectively over control. Moreover, when activity of both the enzymes were compared with the test plants, the 30 µg/ml showed higher activity (37.02% and 51.12%) than plant treated with 20 µg/ml. Overall, the maximum activity of both the enzymes was observed in 30 µg/ml. The result indicates 30 µg/ml ZnO NPs increases the production of ROS which causes oxidative stress in plants. The increase in enzyme activity can also be correlated with the plant condition such as seed germination and plant height which also showed significant decrease over control in 30 µg/ml treatment condition. The similar result was also observed in other plants when treated with Zinc^40,41^. The result suggests the toxic nature of ZnO NPs at higher concentration (30 µg/ml). The effect of ZnO NPs on *Brassica sp*. and other crop plants is summarized in Table 2 and compared with the present result. The present study shows that biosynthesized nanoparticles have the potential to be used as nanofertilizer, provided the effective concentration of the nanoparticles has to be optimized.

**Table 2:** Effect of ZnO NPs on different crop plants.

Plants	NP Concentration in soil/water	NP size(nm)	Effects	References
*Macrotyloma uniflorum*	2–100 mg/L	50	Delayed germination time	^54^
*Fagopyrum esculentum*	10–2,000 mg/L	<50 nm	Decreased the biomass content	^55^
Bean	500 mg/kg	<100 nm	Reduced root growth	^56^
Soybean	500 mg/kg	<100 nm	Ceased seed production	^57^
*Glycine max*	2,000 and 4,000 mg/L	55–70	Genotoxic	^58^
Lettuce	10 mg/kg	41–48	Enhanced the photosynthesis and biomass	^59^
*Cyamopsis tetragonoloba*	10 mg/kg	67	Increased its biomass, shoot-root length, length, chlorophyll content, and total soluble leaf protein	^60^
*Triticum aestivum*	20 mg/L	<100 nm	Increased grain yield and increase in shoot dry weight.	^61^
*Arachis hypogaea* L.	400 and 1000 mg/L	25–100	Improvement in the germination rate and seedling vigor index	^62^
Tomato and egg plants	1.0 mg/mL	38–46	Boost plant defence and yield	^63^
**Effect of ZnO NPs on** ***Brassica*** **species**.				
*Brassica nigra*	500 to 1500 mg/L	<100 nm	Reduced seed germination and seedling growth	^64–66^
*Brassica napus*	10 to 250 mg/L	155 ± 10	Chlorosis at high concentration	^12^
*Brassica juncea*	10–30 μg/ml	11 nm	Increased germination and chlorophyll biosynthesis rate along with low ROS production at 20 μg/ml.	(**Present Study)**
			At 30 μg/ml germination rate, chlorophyll biosynthesis decreases and ROS production increases.	

**Figure 12:**
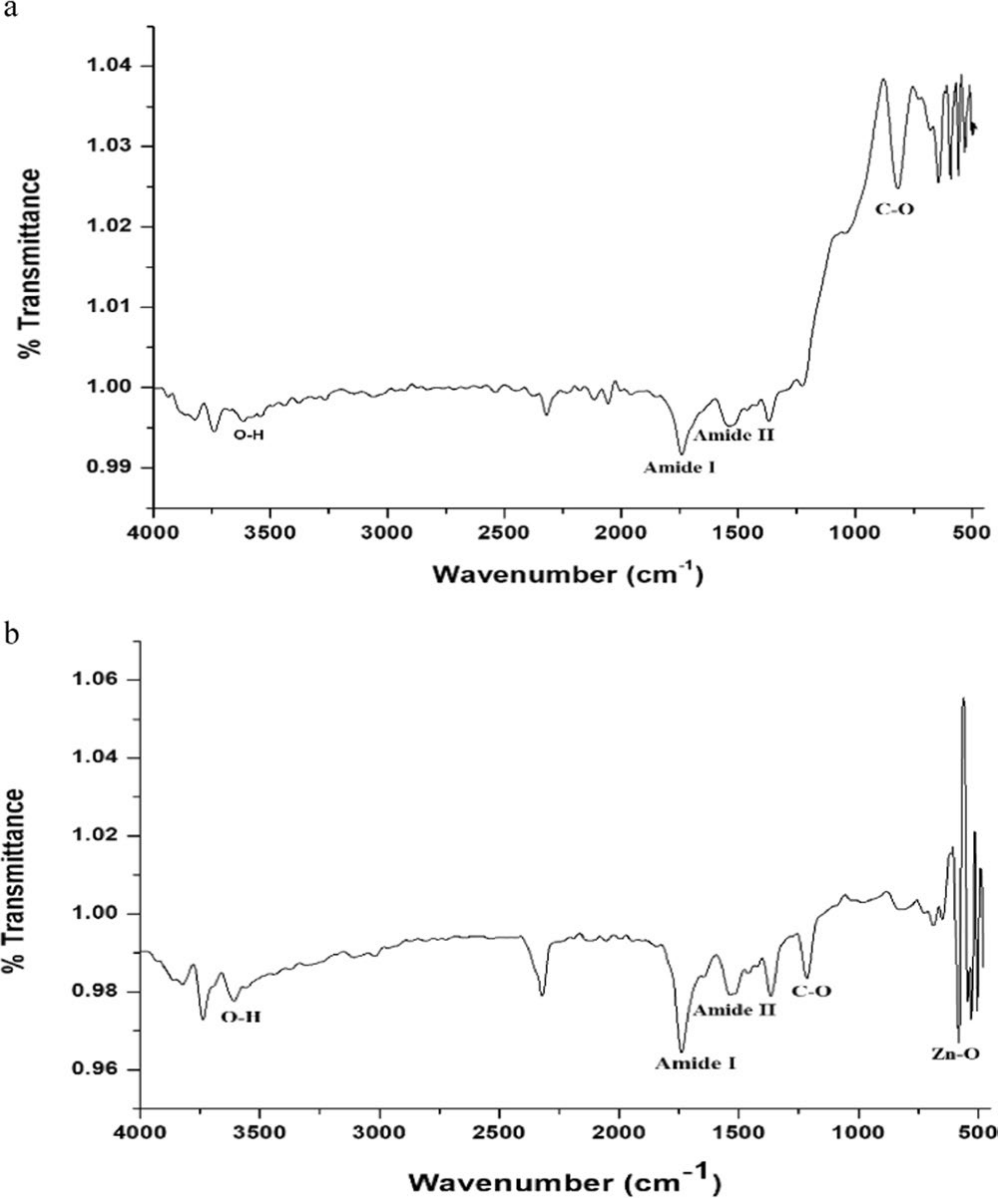
(**a**) FTIR of leaves (Control plant). (**b**) FTIR of Leaves (Test plant 2).

**Figure 13:**
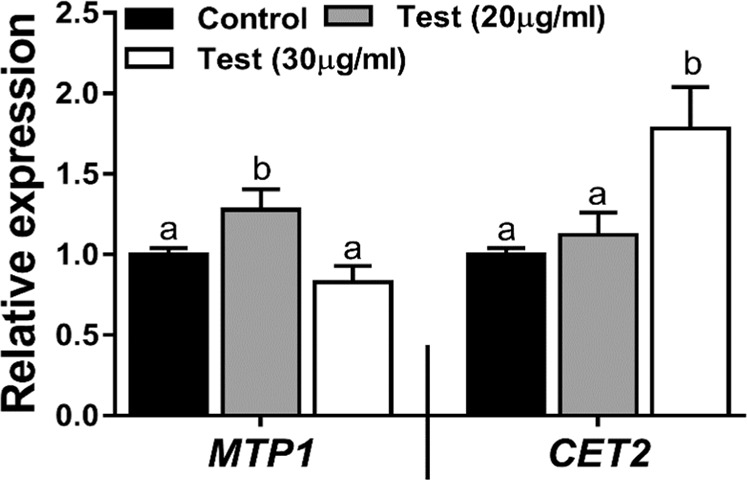
qRT-PCR analysis of MTP1 and CET2 gene expression under 20 μg/ml and 30 μg/ml. The letter a and b indicate the significant difference between the treatments at p < 0.05). Values are means of 4 replicates (n = 4). The endogenous actin of mustard was used as internal control to normalize the expression of genes.

To study the uptake of ZnO NPs by *Brassica* plants, FTIR of leaves was carried out, presence of Zn-O band at 575 cm^−1^ in the spectra revealed the presence of ZnO NPs in the leaves of test plant only, which was absent in control plants as shown in Fig. 12a,b. This may suggests the presence and uptake of ZnO NPs by the leaves of *Brassica*. In general, uptake of metal ion from growth medium, plant require transporter which is expressed in both roots and shoots, plant also require the protein which can sequester the excess metal ion (toxic metal like Zn). To study the mechanism and regulation of ZnO NPs by *Brassica*, expression of two gene the one *BjMTP1* (metal tolerant protein) and second *BjCET2* (cation efflux transporter) were analysed. The key feature of *MTP1* gene is to sequester excess Zn ion into nonreactive component of the cell and *BjCET2* efflux the metal ion out of the cell in case of hyperaccumulator as well as hyper tolerant plants such as *Brassica*^42^. The study reveals that at the concentration of 20 µg/ml there is upregulation of *BjMTP1* and no significant change in *BjCET2* as compared to control, which suggest that ZnO NPs up to 20 µg/ml concentration enhance the tolerance of plants against metal ion. However, downregulation of *BjMTP1* and upregulation of *BjCET2* at the concentration of 30 µg/ml suggest that more than 20 µg/ml concentration is harmful for plants(Fig. 13). Moreover, ZnO NPs (20 µg/ml) show the beneficial role by enhancing the plant germination, growth, chlorophyll content and also regulate the expression of metal transporting genes. Similar results have been reported in the transgenic *Brassica* plant under excess Zinc and Cadmium concentration^43,44^. The present study suggests that enzymes can be used as a template for large scale synthesis of ZnO NPs. Binding site analysis revealed lysine(209), glutamine(158) and tyrosine(155, 238) of alpha amylase interacted with Zinc acetate and helps in the conversion of metal into its nano form. Further studies through molecular dynamic studies may resolve the mystery of how does these interactions change their conformation to reassociate the bonds and produce ZnO NPs. In future, one can design a short peptide containing the amino acids responsible for the synthesis of ZnO NPs, for efficient synthesis of nanoparticles. Size of nanoparticle is one of the prime factor for penetration and movement of nanoparticle into plant tissues, and there are some reports about the maximum dimensions of nanoparticle that plants uptake and facilitate translocation inside the cells is usually below 50 nm^45–47^. As the nanoparticle are of small size in the present study, foliar spray can be done as foliar application of ZnO NPs in semi-arid region can solve the immobilization of element in soil and easy movement of nano-particles through the cuticle of leaves, this can also overcome acute micronutrient deficiency.

## Conclusion

In summary, ZnO NPs were synthesised using alpha amylase enzyme for the first time by the reduction of Zinc acetate dehydrates. The size of ZnO NPs were in the range of 11 nm and spherical in shape. The synthesis method is simple and can be easily scaled. The synthesized nanoparticles enhances the growth and germination of *Brassica juncea* plant, thus can be used as nanofertilizers. Future studies can be carried out in control field trials with *Brassica* and other crops in order to validate our result. The role of ZnO NPs as nano pesticide can also be studied. The risks of nano fertilizers if carefully examined can lead to a sustainable intensification in the field of agriculture.

## Materials and methods

### Chemical and reagents

Zinc acetate dihydrate [(CH_3_COO)_2_Zn.2H_2_O] was purchased from Merck, India. Indian mustard seeds (*Brassica juncea* L.) var. Varuna were purchased from IARI, PUSA, New Delhi, India. Alpha amylase enzyme and RT-PCR kit were purchased from Hi-media (India). Trizol reagent, Nitro blue tetrazolium (NBT), Hoagland’s Media salt (HM) and all other chemicals were purchased from Sigma Aldrich, Life Science.

### Synthesis of ZnO NPs

ZnO NPs was synthesized by incubating 20 ml of alpha amylase (1 mg/ml in distilled water) and 80 ml of freshly prepared aqueous solution of 0.02 M Zinc acetate dihydrate at 25 °C. Appropriate aliquots were withdrawn after regular interval of time and the synthesis was checked by using UV-VIS spectroscopy. After 2 h the reaction mixture was centrifuged at 3000 × g for 10 min and the pellet containing ZnO NPs were washed with distilled water followed by drying. After drying the nanoparticles were used for further characterization. The parametrs like optimum concentration of enzyme and pH were also optimized and synthesis done as above. A control was run in which only 0.02M Zinc acetate dihydrate solution was taken without the enzyme alpha amylase and the spectra were recorded after regular intervals of time.

### Characterization of ZnO NPs

#### UV-Vis spectroscopy

ZnO NPs was initially characterised by using UV-Visible spectroscopy. 1 ml of aliquot was taken out from the solution during the synthesis of ZnO NPs and spectroscopic measurement was run at the wavelength of 250 to 650 nm.

#### Dynamic Light Scattering (DLS)

DLS measurement was carried out using Malvern Zetasizer Nano ZS. All the analysis was done at 25 °C for 10 cycles. ZnO NPs at the concentration of 1 mg/ml in distilled water was used as sample for DLS study.

#### X ray diffraction (XRD)

XRD analysis was performed on Bruker D8 advance diffractometer over a wide range of Bragg angles (20° ≤ 2θ ≤ 80°) as described by Razi *et al*.^48^.

#### Transmission Electron Microscopy (TEM)

TEM analysis of dried ZnO NPs was analysed on JEOL, F2100 instrument as described earlier^48^. An EDX (Model EVO-40, ZEISS) spectrum was used for elemental analysis.

#### Fourier-transform infrared (FTIR) spectroscopy

ZnO NPs in the form of powder was used for analysis in FTIR. FTIR spectra were recorded with a Shimadzu, FTIR between 4000 to 500 cm^−1^, with a resolution of 4 cm^−1^.

### Binding site analysis of α-amylase to Zinc acetate through in silico docking with GOLD

An in silico docking experiment was carried out to determine the probable binding residues of α-amylase with zinc. The protein structure was taken from RCSB-Protein Data Bank (PDB ID-3VX0 solved at a resolution of 1.5 Å). The original protein structure was manually curated to remove Gadolinium ions, Calcium ion, HOH (water) molecules and NAG(N-acetyl-D-glucosamine) molecule. Energy minimization step were carried out using SPDBV(Swiss PDB Viewer). The ligand structure coordinate file for Zinc acetate was downloaded from PubChem database available with the ID Structure2D_CID_11192.

GOLD 5.2.2 was used for docking, the moderate flexibility of atomic bonds and rotational angles in protein backbone and 10 GA runs per ligand molecule was checked. Results obtained were extracted as a combined list in CSV format to consider every feature of their interactions. The original files were studied further using PyMol provided by Schrodinger on academic license. Since, total number of probable solutions were only three, all complexes were studied extensively to recognize the pattern of Protein-Ligand interaction and to identify the responsible amino acids with their respective distances.

### Effect of ZnO NPs on seed germination and growth of plant

#### Treatment conditions

Mustard (*B. juncea* L.) seeds were surface sterilized in 1% (v/v) sodium hypochlorite for 15 min and washed thoroughly with distilled water. Sterilized seed were soaked in deionized water (DW) for overnight and then transferred to PVC cup (equal no. of seeds were spread in each cup) containing 10% Hoagland medium (HM). After one day, the seeds were then subjected to treatment. The treatment includes, (1) control having 5% HM only (2) ZnO NPs 10 μg/ml (Test 1), 20 μg/ml (Test 2) and 30 μg/ml (Test 3) dissolved in 5% HM in DW water. Once the seeds germinated, the plantlets were transferred to controlled growth chamber, provided 16-hrs photoperiod and 25 ± 2 ^°^C temperature for 7 days. The same treatment as that of seeds was provided to the plantlets, 10% HM was used for plantlets. The treatment solution was replaced after every 2 days interval in order to avoid nutrient limitation. After 7 days of treatment, plants were harvested and frozen immediately in liquid nitrogen for further experiment. Seed germination experiment were carried out in five replicates.

#### Morphological analysis

The rate of seed germination of *Brassica* var. Varuna, treated with different concentration (i.e 10–30 μg/ml) of ZnO NPs was calculated after 3 days of treatment. The percentage seed germination was computed using the formula i.e. (total number of germinated seeds / total number of seed per cup) X 100%. The seeds were considered germinated when both plumule and radicle are come out from their junction. Further the shoot and root lengths were measured by taking 7 days old treated plants using scale.

#### Chlorophyll (Chl) estimation

Plants treated with ZnO NPs after one week were estimated for chlorophyll content as described previously^49^. 50 mg of *Brassica juncea* leaves was cut into fine pieces and crushed properly. 1.0 mL Dimethyl sulfoxide (DMSO) was added to the extract and the sample was mixed properly using a vortex for 30 s. The sample was centrifuged at 15000 x g for 5 min followed by which supernatant was removed and the pellet was mixed with 1.0 mL of DMSO to re-extract. The extract was then transferred to glass cuvette and spectrophotometrically analysed at 649 and 665 nm with a resolution of 1 nm.

Following formula was used to estimate total chlorophyll (a + b).

Amount of chl a, C_a_ = 12.19A_665_ − 3.45A_649_

Amount of chl b, C_b_ = 21.99 A_649_ − 5.32 A_665_

#### Enzyme extraction and assay

To extract total enzyme, fresh sample (200 mg) was homogenized in phosphate buffer as described by Mostofa *et al*.^50^. Superoxide dismutase (SOD) activity was analysed according to Dhindsa *et al*.^51^. The assay is based on the photochemical reduction inhibition of NBT (nitro blue tetrazolium). The assay mixture (1 mL) contain enzyme extract (100 µL) phosphate buffer (0.05), methionine (2 mM), EDTA (2.5 mM), Riboflavin (10 mM), Na_2_CO_3_ (1 M) and NBT (2.25 mM). Superoxide formation was observed at 560 nm and enzyme activity of SOD was measured as EU min^−1^ mg^−1^ protein. Catalase (CAT) (EC 1.11.1.6) activity was measured according to Aebi with some modifications^52^. The assay mixture contained enzyme extract (50 μL), reaction buffer (0.1 M phosphate buffer, pH 7.8) and H_2_O_2_ (10 mM) prepared in phosphate buffer (pH 7.8) Decrease in absorbance was noted after every 1 min for up to 3 min.

#### FTIR based localization studies of ZnO nanoparticles in the leaves of Brassica plant

For FTIR, the leaves (50 mg) of the plant (both Test 2 and control) were chopped and crushed separately (using methanol as solvent) and dried to form powder. The powdered leaf was used to record the FTIR spectra as described^53^.

#### RNA extraction and Quantitative Real-Time PCR (qRT-PCR) Analysis

Trizol reagent (Sigma Aldrich Life Science) was used for the isolation of Total RNA from 0.1 g leaf of *Brassica* plants (7 Day old control and treated plant). Nanodrop-spectrophotometer was used for RNA quantification and further, assessed on to 1.2% agarose gel. cDNA synthesis was carried out by using RevertAid H Minus First Strand cDNA synthesis kit (Thermo fisher). Approximately, 2 µg of total RNA was used for cDNA synthesis. The qRT-PCR analysis was done with SYBR Green as described previously by Pandey *et al*.^54^. Samples are analysed in triplicate, and relative expression was analysed by using 2^−(ΔΔCt)^ method described by Livak *et al*.^55^. The endogenous actin of mustard was considered as internal control, the primers used for analysis are listed in Table 3.

**Table 3:** List of primer used for qRT-PCR analysis.

Key Genes	Source	Primer Sequence 5′-3′ direction
*Actin*	*Brassica juncea*	F-TAACAGAGAGAAGATGACTC R-CAGAGTCAAGCACAATAC
*MTP1*	*Brassica juncea*	F-GTGACTGTTACCACACAT R-CTCTTCCTCTTCTCTGAT
*CET2*	*Brassica juncea*	F-CTGTCATCTGGTACAAAC R -TGTACTCTCCATCAACAC

## Statistical analysis

The result presented for seed germination and root-shoot length are the mean from five replicates ± SD. Statistical analyses of the qPCR result are mean from three replicates ± SD. One-way ANOVA or independent-samples t-test was conducted to analyse significant differences. In case of the rest of the data statistical significance was taken as P < 0.05. Data have been presented as mean + SD.
